# Morphological characterization of fullerene–androsterone conjugates

**DOI:** 10.3762/bjnano.5.43

**Published:** 2014-03-28

**Authors:** Alberto Ruiz, Margarita Suárez, Nazario Martin, Fernando Albericio, Hortensia Rodríguez

**Affiliations:** 1 Laboratorio de Síntesis Orgánica, Facultad de Química, Universidad de La Habana, 10400 La Habana, Cuba; 2 Departamento de Química Orgánica I, Facultad de Ciencias Químicas, Universidad Complutense de Madrid 28040 Madrid, Spain; 3 Institute for Research in Biomedicine, Barcelona Science Park, Baldiri Reixac 10, 08028-Barcelona, Spain; 4 Centre on Bioengineering, Biomaterials and Nanomedicine, PCB, 08028-Barcelona, Spain; 5 Department of Organic Chemistry, University of Barcelona, 08028-Barcelona, Spain; 6 School of Chemistry, University of KwaZulu-Natal, Durban 400l, South Africa

**Keywords:** androsterone, dynamic light scattering, fullerene, transmission electron microscopy

## Abstract

Here we report on the self-organization characteristics in water of two diastereomer pairs of fullerene–androsterone hybrids that have the hydrophobic C_60_ appendage in the A and D ring of the androsterone moiety, respectively. The morphology and particle size in aqueous solution were determined by transmission electron microscopy (TEM) and dynamic light scattering (DLS), with satisfactory agreement between both techniques. In general, these fullerene derivatives are shown to organize into spherical nano-scale structures with diameters in the ranges of 10–20 and 30–50 nm, respectively.

## Introduction

Since the discovery of [60]fullerene [1], the efforts of the scientific community have been focused on the preparation of suitably functionalized fullerene derivatives with physical and biological properties interesting for biomedicine and materials science [2]. The covalent linkage of C_60_ to moieties, such as porphyrins [3], anionic polymethine cyanine [4], and other bioactive molecules such as amino acids [5], peptides [6–7], nucleotides, sugars and steroids [8–9], have allowed the solubilization of these hybrid derivatives in aqueous media, thus enhancing certain biological activities. For the potential use of C_60_ derivatives as drug delivery systems, the size of the particles is important. In general, fast drug release was observed for small particles, although these particles tended to aggregate. For this reason, a compromise between the size and the stability of dispersion is necessary in order to develop efficient systems [10]. On the other hand, androsterone [11] is an important and well-known metabolite of testosterone, the most prevalent androgen in males. The conjugation of steroids to other chemically or biologically relevant molecules is a common approach in the search for new biomedical and chemical applications. The coupling of C_60_ with a steroid changes the physicochemical properties of this molecule, improving its solubility and biocompatibility, thus facilitating further bioactivity studies [12]. The morphology and aggregation properties of some fullerene derivatives have been previously established. Brettreich et al., demonstrated that hexa-adducts of [60]fullerene functionalized with both hydrophobic and hydrophilic moieties, form spherical vesicles known as “buckysomes” in aqueous media [13]. The same behavior has also been observed by Conyers et al. [14] and Martin et al. [15] for other fullerene derivatives. As representative examples, the pentamethyl[60]fullerene salt Me_5_C_60_K and the pentaphenyl[60]fullerene salt Ph_5_C_60_K have a tendency to form closed submicron spheres [16–17]. To the best of our knowledge, all reported C_60_–steroid derivatives have been spectroscopically characterized. However, the aggregation properties of these entities in water have not been studied to date.

Here we report on the morphological characterization of four fullerene–androsterone conjugates in water. The morphology and particle size of the C_60_–androsterone hybrids in aqueous solution were determined by transmission electron microscopy (TEM) and dynamic light scattering (DLS).

## Results and Discussion

**Figure 1:**
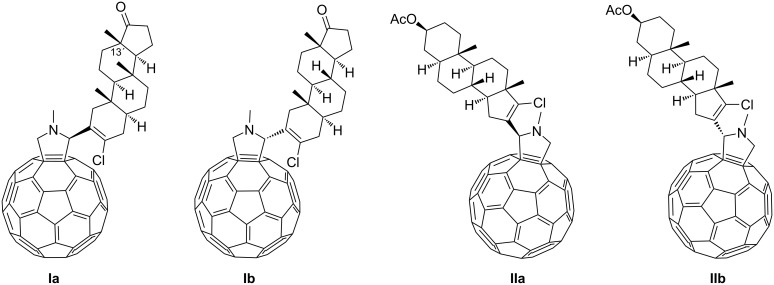
Chemical structure of the different C_60_–androsterone hybrids.

The major drawback of [60]fullerene is its low solubility in water and others solvents, except toluene or *o*-DCB. The water-soluble C_60_–androsterone conjugates **Ia**, **Ib**, **IIa** and **IIb** (Figure 1) were prepared in a multistep synthetic procedure by following the recently reported methodology [18]. The C_60_ unit was covalently connected to the steroid moiety via a Prato reaction to obtain a diasteromeric mixture of **I**, with the hydrophobic C_60_ appendage in the A ring of the steroid moiety (**Ia** and **Ib**), and **II**, in which the steroid D ring was modified (**IIa** and **IIb**). These two mixtures of diastereomers were easily separated by flash chromatography, which allowed pure derivatives to be obtained as stable brown solids [17]. The aggregation properties of C_60_–androsterone conjugates (**Ia,b** and **IIa,b**) were subsequently studied by TEM and DLS techniques.

### Transmission electron microscopy

**Figure 2:**
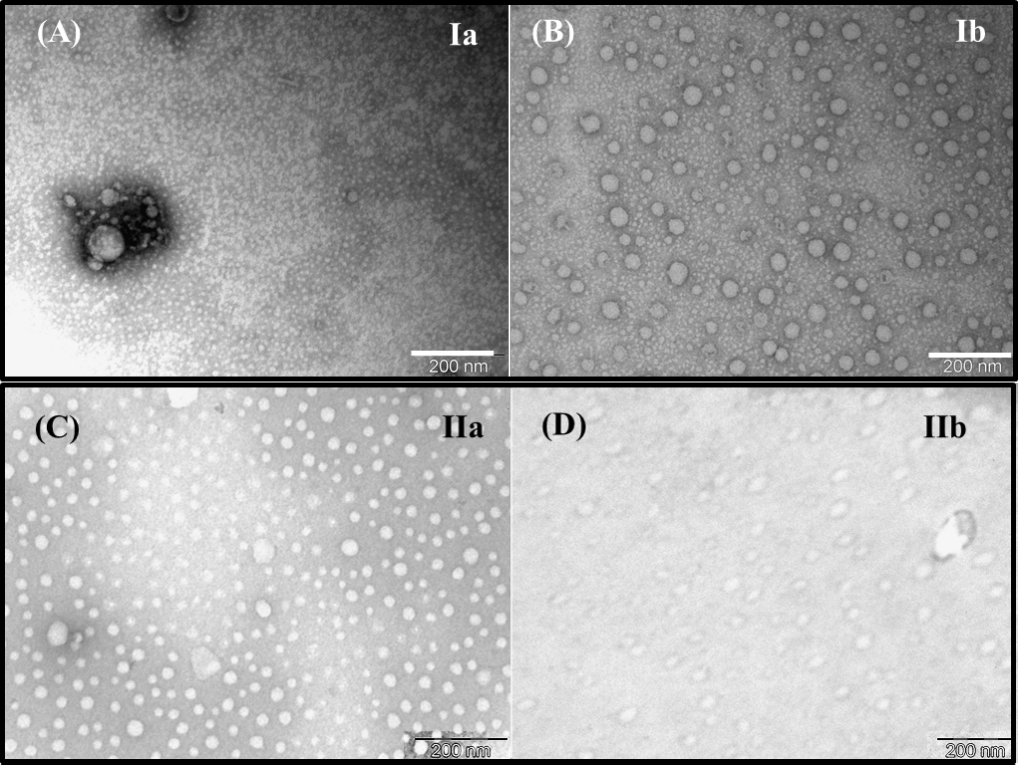
Representatives uranyl acetate negative stain transmission electron micrographs (TEM) of fullerene–androsterone conjugates.

Uranyl acetate negative stain TEM was performed on the four C_60_–steroid derivatives (**Ia,b** and **IIa,b**) in aqueous solution. In general, the TEM imaging of the C_60_–androsterone nanoparticles showed that they are polydisperse and mainly spherical, with slight angular features (Figure 2 and Supporting Information File 1). In order to ensure a representative analysis of each sample, several areas of the grids were observed. The two diastereomeric pairs (**Ia,b** and **IIa,b**) showed spherical self-assembly due to the non-covalent interactions present in these compounds, i.e., van der Waals forces, hydrogen bonding, hydrophilic/hydrophobic interactions, π–π stacking interactions, and donor–acceptor interactions. The aggregation behavior of these compounds was associated to the hydrophobic nature of the C_60_ core as the main attractive force, furthermore, the presence of different polar groups, carbonyl and acetoxy group for **Ia,b** and **IIa,b** respectively, in the appendage of these monoadducts, may be responsible of the difference size between both diastereomeric pairs, considering the different strength of the hydrogen bonds between these groups and water. Similar behavior has been observed in C_60_ monoadducts with one or two hydrophilic heads [19–22].

**Figure 3:**
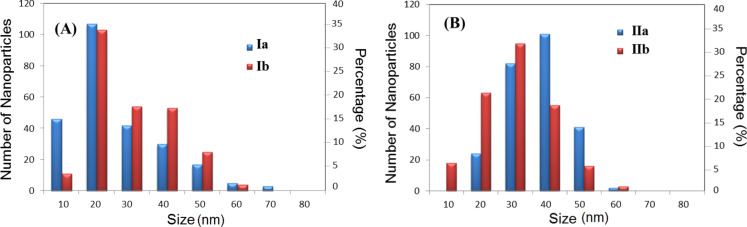
Size distributions of 250 nanoparticles of C_60_–androsterone (A) **Ia,b** and (B) **IIa,b** adsorbed on the TEM grids.

For each sample, the diameters of 250 nanoparticles were determined from the TEM images by using the ImageJ software. Figure 3 shows the size distribution of the 250 nanoparticles for both diastereomeric pairs, with each bar representing a diameter range of 10 nm. The fullerene–androsterone conjugates **Ia,b** predominantly showed nanoparticles in the 11–20 nm size range, about 15% of the population of **Ia** nanoparticles were in 5–10 nm range. Furthermore, larger aggregates from 21 to 60 nm were seen, and isolated nanoparticles that measure 71–80 nm were occasionally observed for diasteromer **Ia**. Particles larger than 70 nm were not observed for the diasteromer **Ib** (Figure 3A). The other diasteromer pairs **IIa,b** showed slightly larger nanoparticles than **Ia,b**, as well as a more heterogeneous population distribution, although with a predominant size in the range of 31–50 nm (Figure 3B).

The size and shape of these aggregates can be attributed to the hydrophilic moiety that is linked to [60]fullerene in the C_60_–androsterone derivatives (**Ia,b** and **IIa,b**). The steroid linked to fullerene promotes hydrophilicity and steric repulsion to offset the C_60_ fragment, and therefore these derivatives tend to self-organize as unilamellar vesicles, and form spherical aggregates with a variety of sizes, but with a well-defined spherical shape. In general, when the C_60_ moiety was attached into the D rind (**IIa,b**) instead of the A ring (**Ia,b**) of the androsterone moiety, larger aggregates were obtained. These results are a consequence of the difference in hydrophilicity of the androsterone moieties in both diasteromeric pairs. The presence of a carbonyl group in **Ia,b** instead of a less polar group such as acetoxy in **IIa,b**, promoted the formation of smaller vesicles.

### Dynamic light scattering

**Figure 4:**
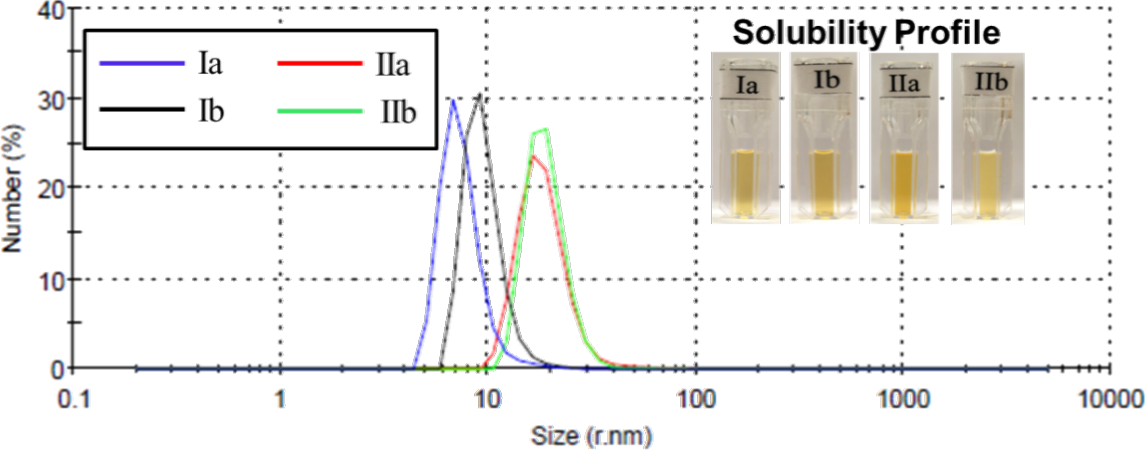
Histogram analyses of the particle size distribution by number, and solubility profile in water at 0.1 mg·mL^−1^ of fullerene–steroid derivatives **Ia,b** and **IIa,b**.

DLS measurements gave further information about the particle size of the C_60_–androsterone conjugates. The concentration range studied was 0.1–0.4 mg·mL^−1^. The histograms of the four C_60_–androsterone derivatives **Ia,b** and **IIa,b** in Figure 4 show the average particle size distribution in water at 0.1 mg·mL^−1^, in which the particles of 5–12 nm and a more broad range of 12–26 nm of hydrodynamic radius are shown for **Ia,b** and **IIa,b**, respectively. The four samples analyzed had polydispersity index values of 0.393 to 0.454, which are consistent with polydisperse samples.

**Figure 5:**
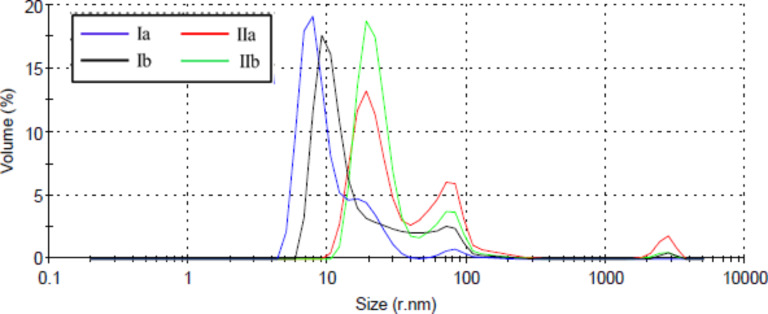
Histogram analyses of the particle size distribution by volume of fullerene–steroid derivatives **Ia,b** and **IIa,b**.

The histograms of particle size distribution by volume at the same concentration, of the four C_60_–androsterone derivatives **Ia,b** and **IIa,b** in Figure 5 show at least three distinct populations of particles. In general, the derivatives **II** showed mean populations of particles with slightly higher size (*r* = 14–30 nm) than derivatives **I** (*r* = 6–22 nm). Similar DLS results were obtained for the C_60_–androsterone conjugates (**Ia,b** and **IIa,b**) at 0.4 mg·mL^−1^ (see Supporting Information File 1).

Although DLS and TEM techniques showed the same trend to the aggregation behaviour of both diasteromeric pairs **Ia,b** and **IIa,b**, slightly but systematic differences in vesicle size were detected. The dynamic light scattering (DLS) measures the hydrodynamic diameter of the particle including the solvation layers, while TEM gives the most direct information about the size distribution and shapes of the primary particles. DLS and TEM have different basis and can lead to discrepancies in sizing.

## Conclusion

The molecular self-assembly characteristics of C_60_–androsterone derivatives **Ia,b** and **IIa,b** in water involve the formation of nano-sized spherical vesicles with most particles falling in the size ranges of 11–20 nm and 31–50 nm for **Ia,b** and **IIa,b**, respectively, as estimated by TEM. DLS measurements showed that all samples formed aggregates in water. The tendency of the sphere size distribution results is in good agreement with TEM micrographs. Both TEM and DLS results revealed that **IIa,b** derivatives are slightly larger. It should be noted that the introduction of the C_60_ cage in a different position on the androsterone moiety induces morphologically distinct nanostructures in water. Thus these particles have the same shape but differ in size.

## Experimental

The fullerene–androsterone conjugates (**Ia,b** and **IIa,b**) were synthesized as previously described [18]. The nanoparticle solutions were made by dissolving the corresponding sample (10 mg) in H_2_O (10 mL) with ultrasonication for 2 h, followed by centrifugation (10 min, 3000 rpm) and the transparent brown suspension was transferred to a clean vessel. The resulting suspension was analysed by TEM and DLS.

### Transmission electron microscopy

The aggregates were visualized using uranyl acetate negative staining. One drop of the clear solution was transferred to a TEM grid (copper grid, 3.0 mm, 200 mesh, coated with Formvar film), together with a drop of uranyl acetate (2% water solution) for 1 min, and allowed to dry. Analysis of stained grids was performed with a JEOL JEM 2100 (Tokyo, Japan) Transmission Electron Microscope.

### Dynamic light scattering

Light scattering experiments were performed on a Zetasizer Nano S ZS90 (Malvern Instruments, USA). Data were collected at 25 °C by monitoring the scattered light intensity. The hydrodynamic radius and the polydispersity index of the samples were calculated by using the CONTIN analysis software. Each light scattering measurement was performed at least three times.

## Supporting Information

File 1 Additional TEM images and DLS data for the C_60_–androsterone conjugates **Ia,b** and **IIa,b**.
