# Supplementary material for: Morphological characterization of fullerene–androsterone conjugates
Source: Beilstein J Nanotechnol. 2014 Mar 28;5:374–9. doi: 10.3762/bjnano.5.43 (PMC3999864; doi:10.3762/bjnano.5.43)

Supporting Information

for

Morphological characterization of fullerene–androsterone conjugates

Alberto Ruiz[1], Margarita Suárez[1], Nazario Martin[2], Fernando Albericio[* 3,4,5,6] and Hortensia Rodríguez[*3]

Address: [1]Laboratorio de Síntesis Orgánica, Facultad de Química, Universidad de La Habana, 10400 La Habana, Cuba; [2]Departamento de Química Orgánica I, Facultad de Ciencias Químicas, Universidad Complutense de Madrid 28040 Madrid, Spain; [3]Institute for Research in Biomedicine, Barcelona Science Park, Baldiri Reixac 10, 08028-Barcelona, Spain; [4]Centre on Bioengineering, Biomaterials and Nanomedicine, PCB, 08028-Barcelona, Spain; [5]Department of Organic Chemistry, University of Barcelona, 08028-Barcelona, Spain; and [6]School of Chemistry, University of KwaZulu-Natal, Durban 400l, South Africa

Email: Hortensia Rodríguez* - hortensia.rodriguez@irbbarcelona.org;

Fernando Albericio* - fernando.albericio@irbbarcelona.org

* Corresponding author

TEM images and dynamic light scattering data for the C_{60}-androsterone conjugates Ia-b and IIa,b

Uranyl acetate negative stained transmission electron micrographs (TEM) of fullerene-androsterone conjugates Ia-b and IIa-b.

N-Methyl-2-substituted-pyrrolidino[3,4:1,2]-[60]fullerenes (**Ia-b** and **IIa-b**)

N-Methyl-2(R)-(3′-chloro-17′-oxo-(5α-antrostan-2′-en-2-yl)-pyrrolidino[3,4:1,2][60]fullerene (**Ia**)

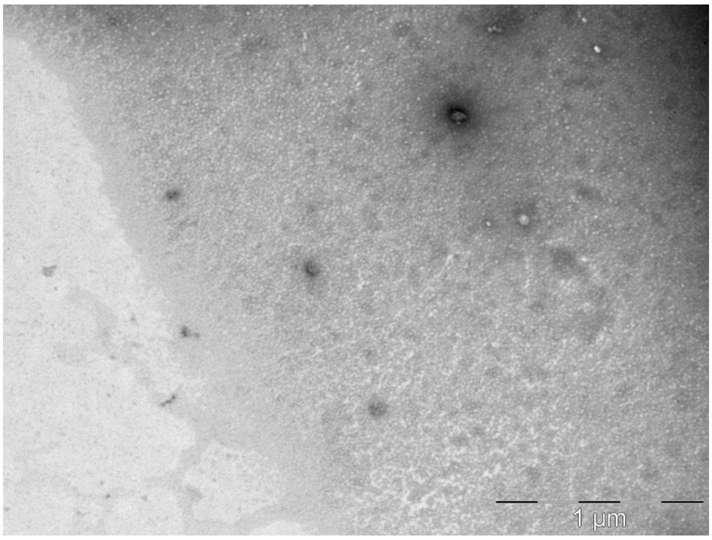

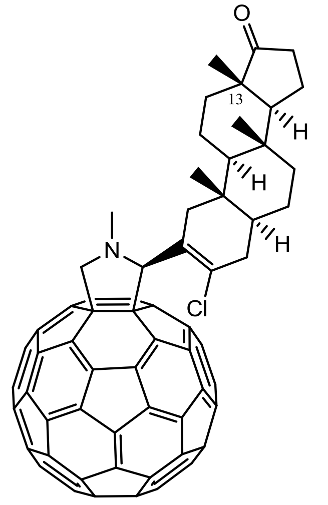

Ia

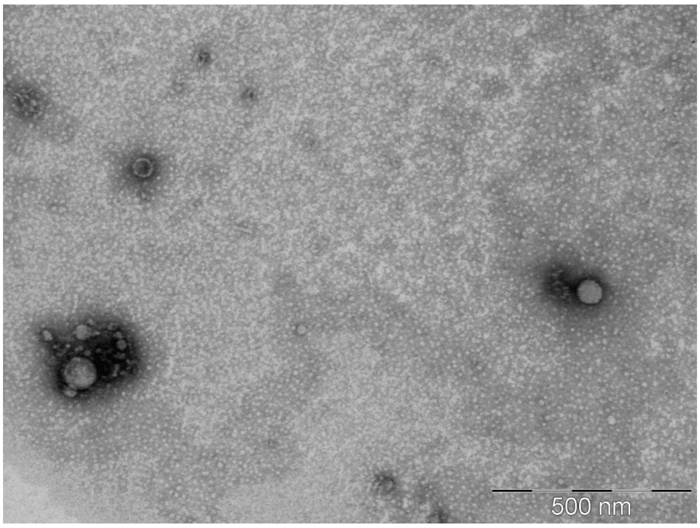

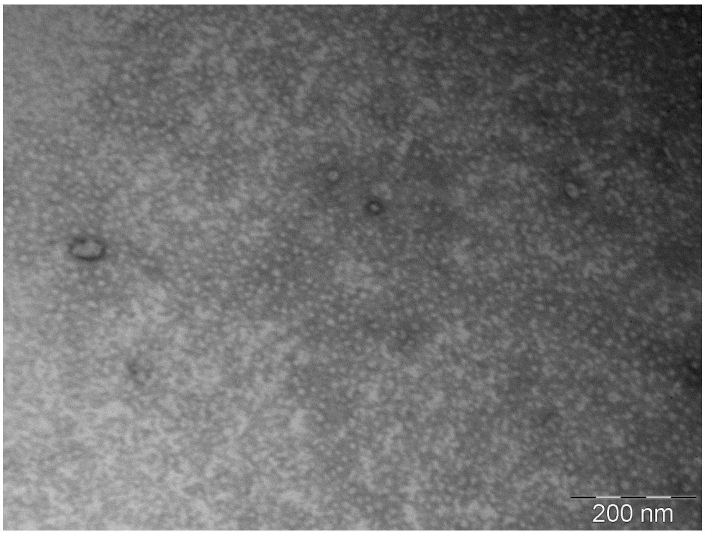

N-Methyl-2(S)-(3′-chloro-17′-oxo-(5α-antrostan-2′-en-2-yl)-pyrrolidino[3,4:1,2][60]fullerene (**Ib**)

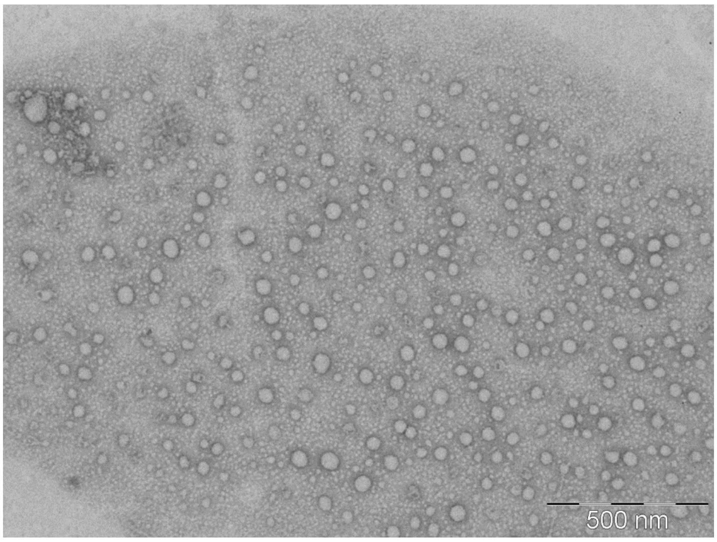

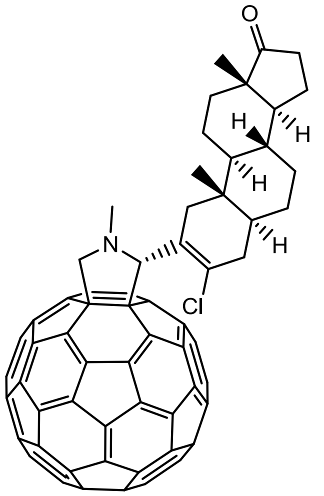

Ib

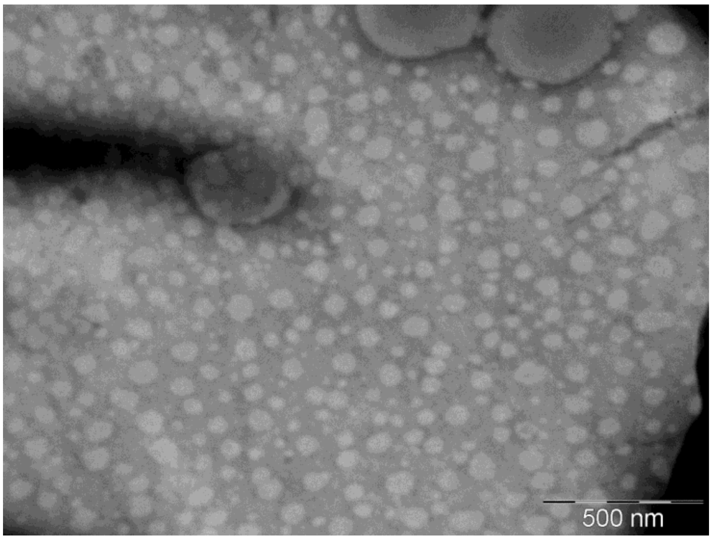

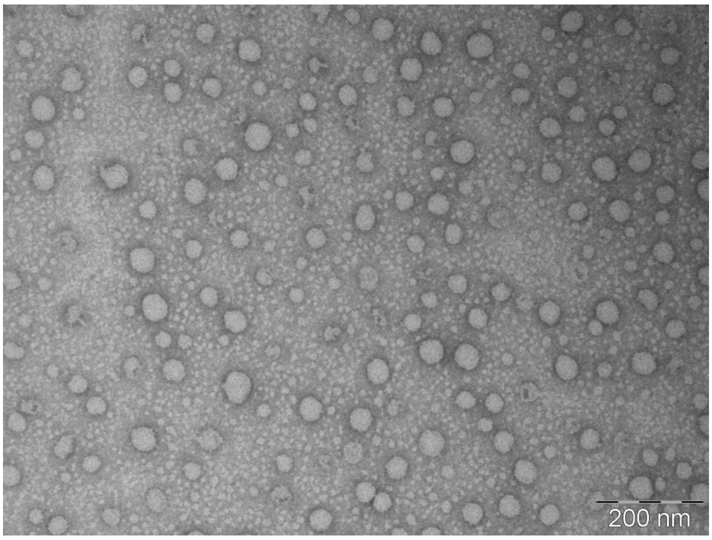

N-Methyl-2(R)-(3′β-acetoxy-17′-chloro-5α-androstan-16-en-2-yl)pyrrolidino[3,4:1,2][60]fullerene (**IIa**)

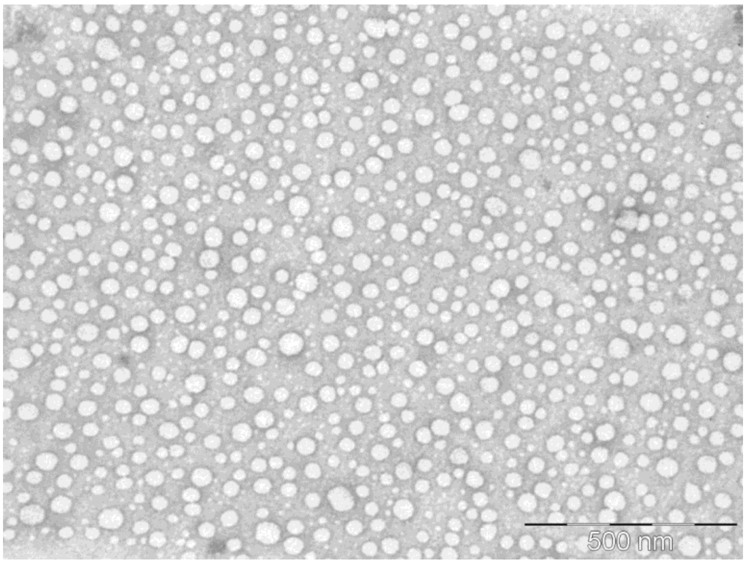

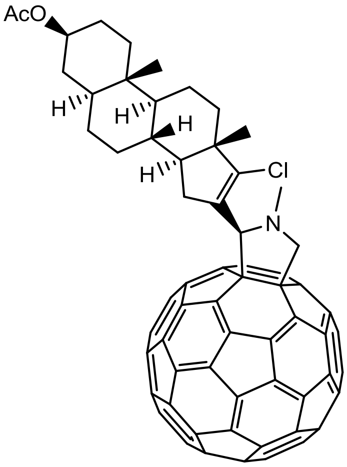

IIa

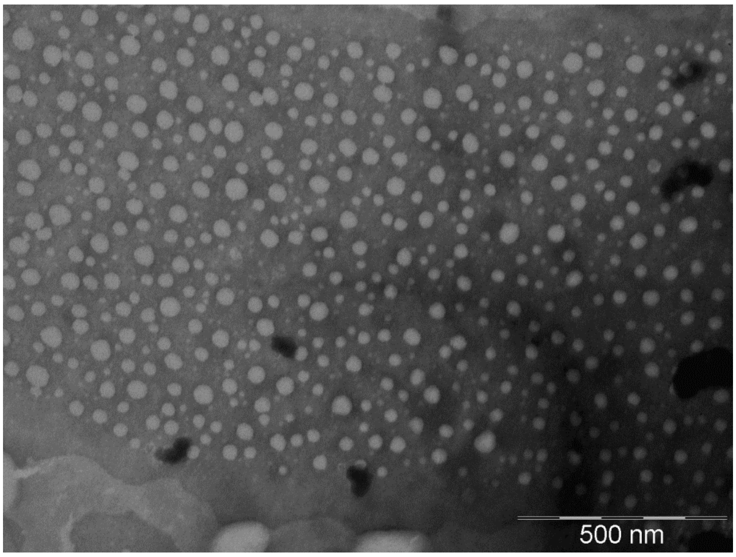

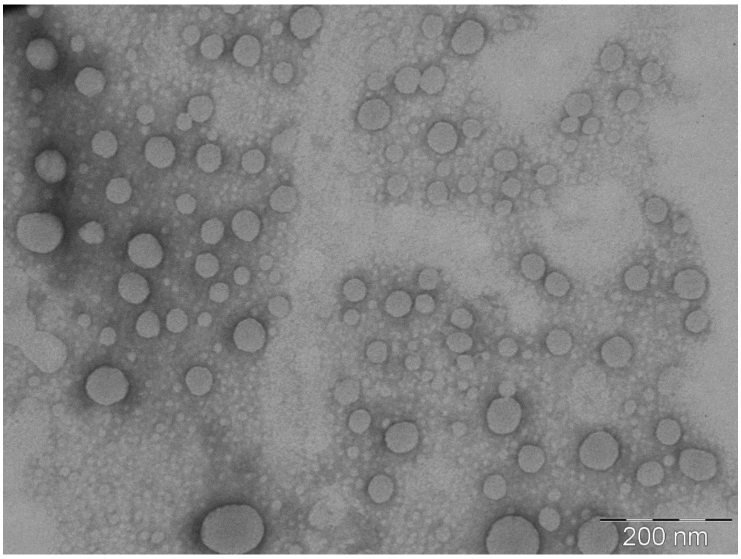

N-Methyl-2(S)-(3′β-acetoxy-17′-chloro-5α-androstan-16-en-2-yl)pyrrolidino[3,4:1,2][60]fullerene (**IIb**)

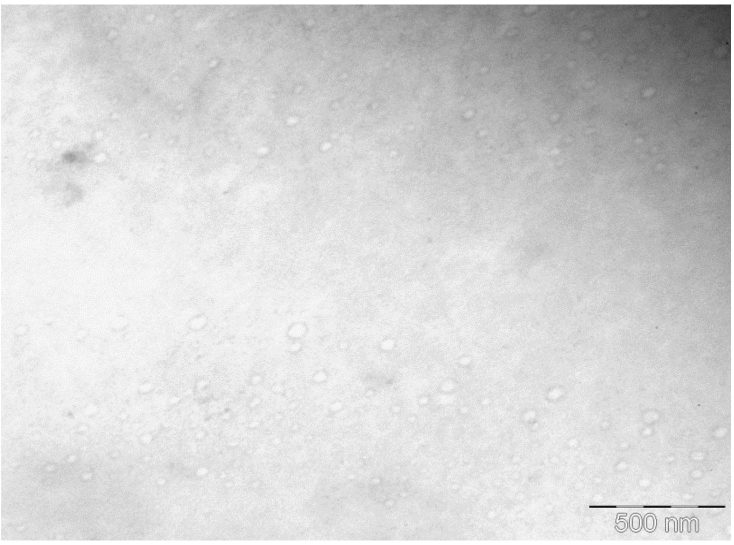

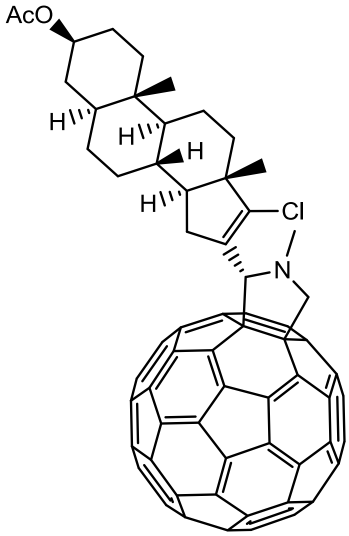

IIb

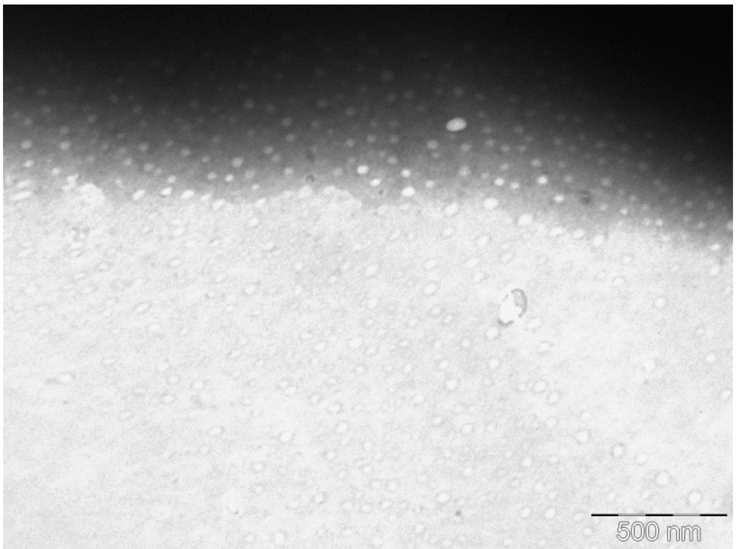

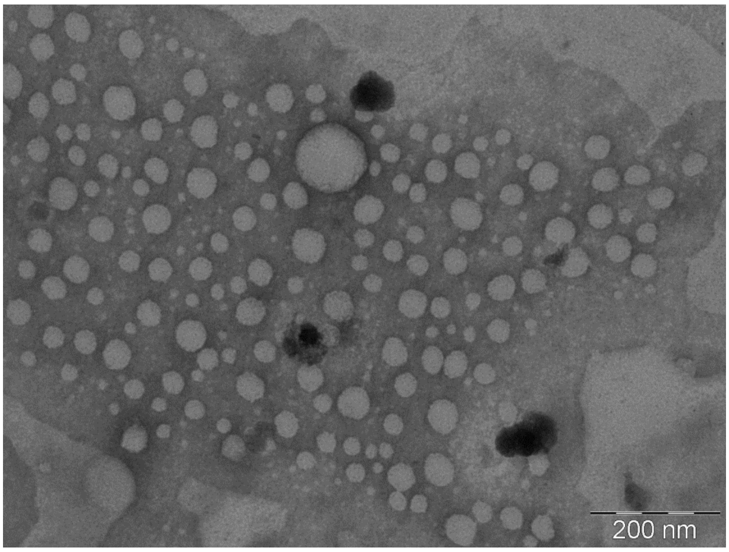

Dynamic Light Scattering data

Histogram analyses of the particle size distribution by number, by volume and by intensity.

N-Methyl-2(R)-(3′-chloro-17′-oxo-(5α-antrostan-2′-en-2-yl)-pyrrolidino[3,4:1,2][60]fullerene (**Ia**)
Concentration: 0.1 mgmL^{-1}

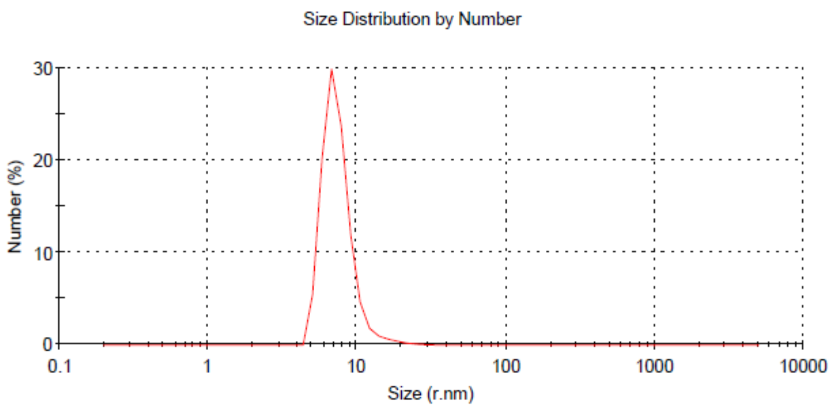

PdI: 0.393

Intercept: 0.960

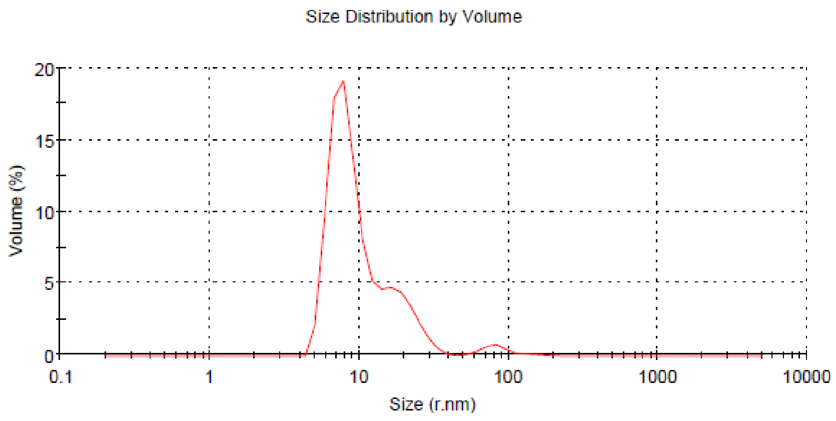

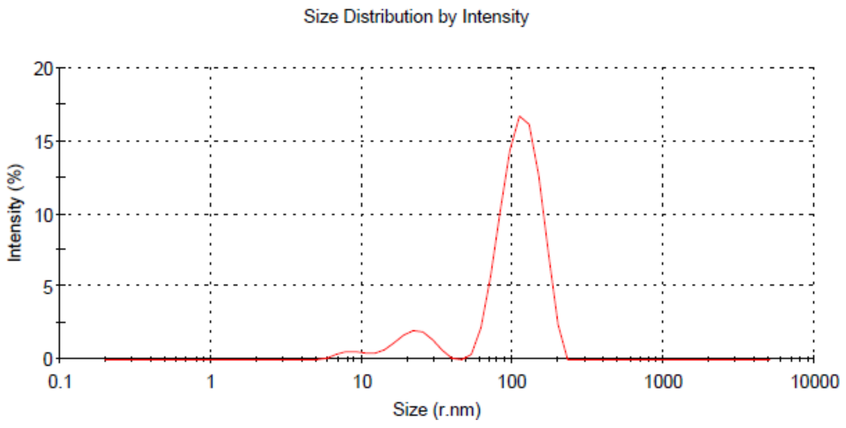

Concentration: 0.4 mgmL^{-1}

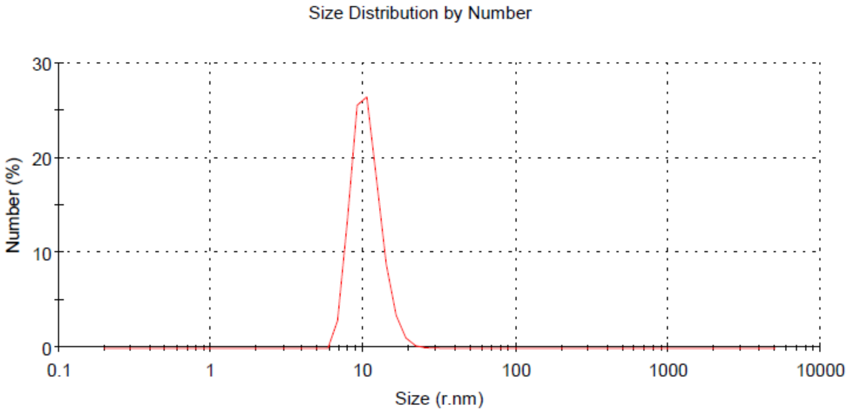

PdI: 0.494

Intercept: 0.941

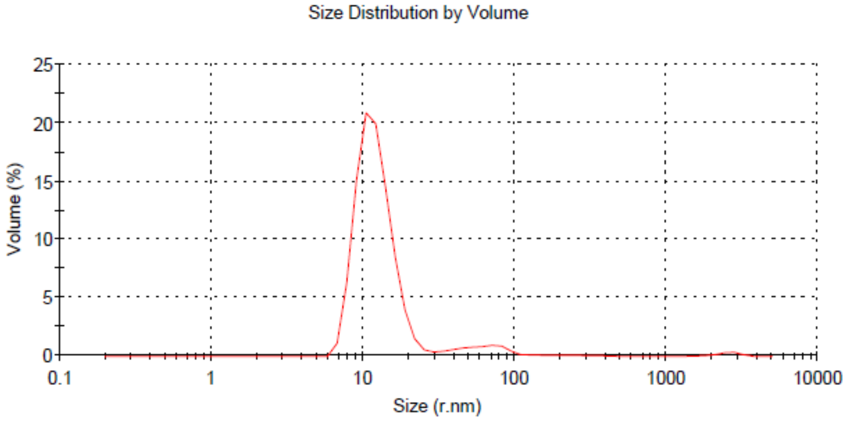

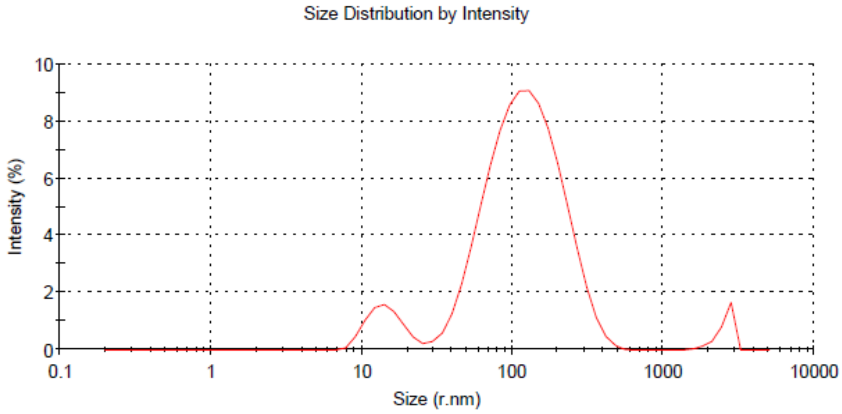

N-Methyl-2(S)-(3′-chloro-17′-oxo-(5α-antrostan-2′-en-2-yl)-pyrrolidino[3,4:1,2][60]fullerene (**Ib**)
Concentration: 0.1 mgmL^{-1}

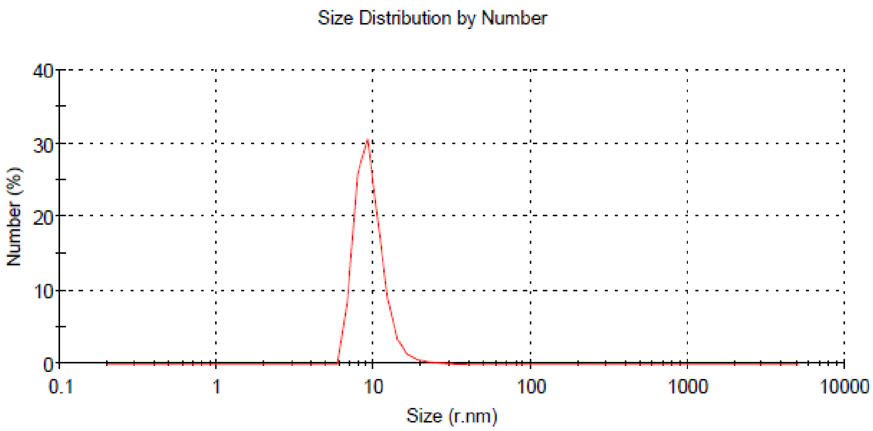

PdI: 0.454

Intercept: 0.941

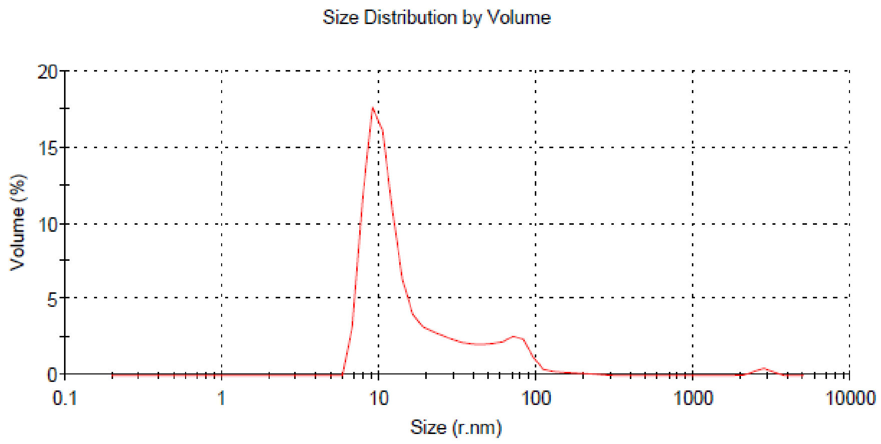

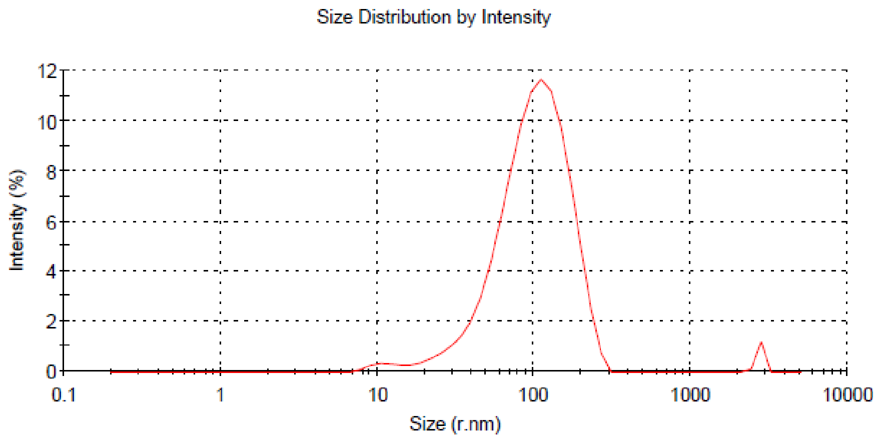

Concentration: 0.4 mgmL^{-1}

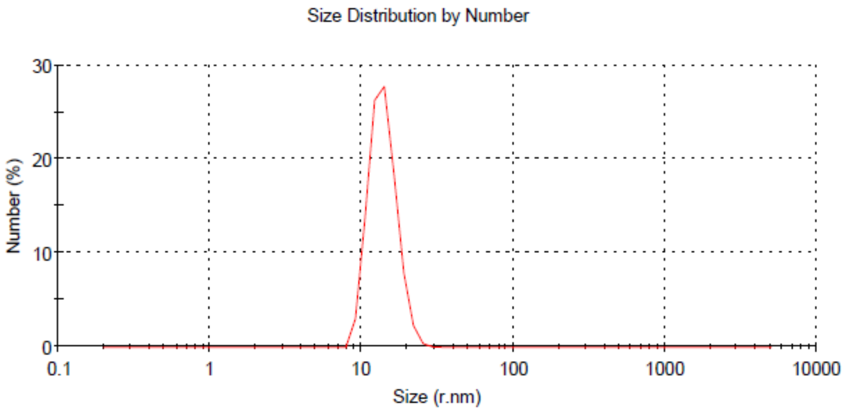

PdI: 0.339

Intercept: 0.930

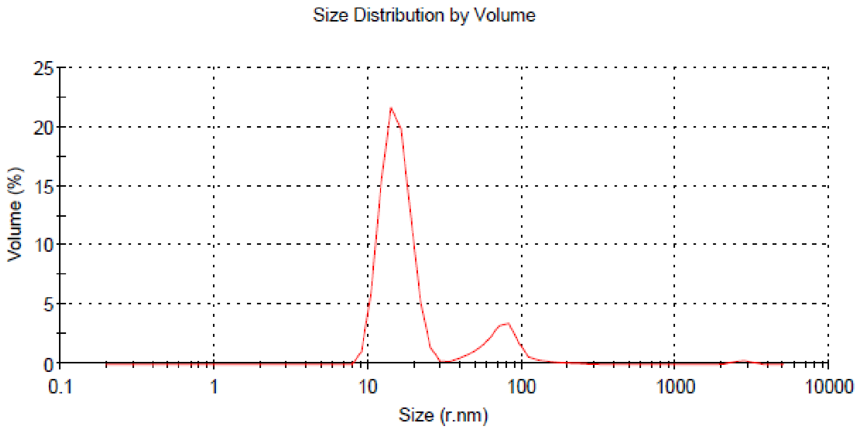

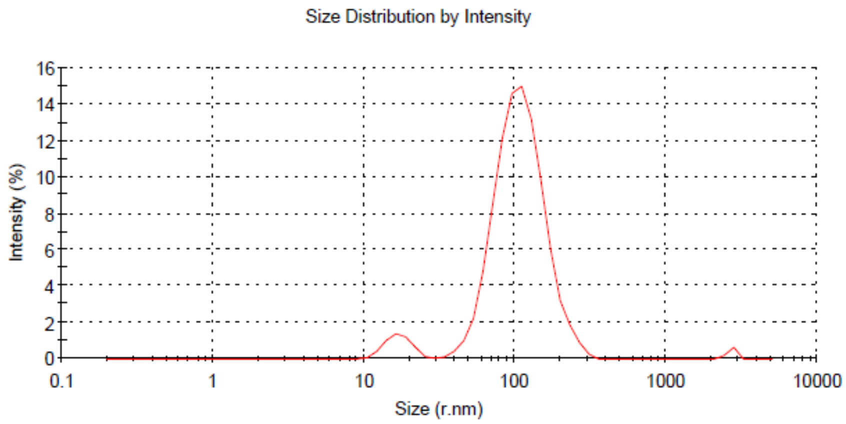

N-Methyl-2(R)-(3′β-acetoxy-17′-chloro-5α-androstan-16-en-2-yl)pyrrolidino[3,4:1,2][60]fullerene (**IIa**)

Concentration: 0.1 mgmL^{-1}

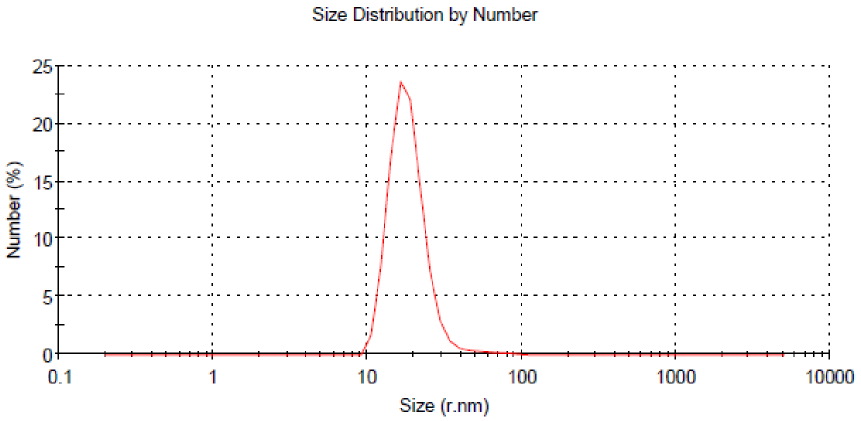

PdI: 0.409

Intercept: 0.952

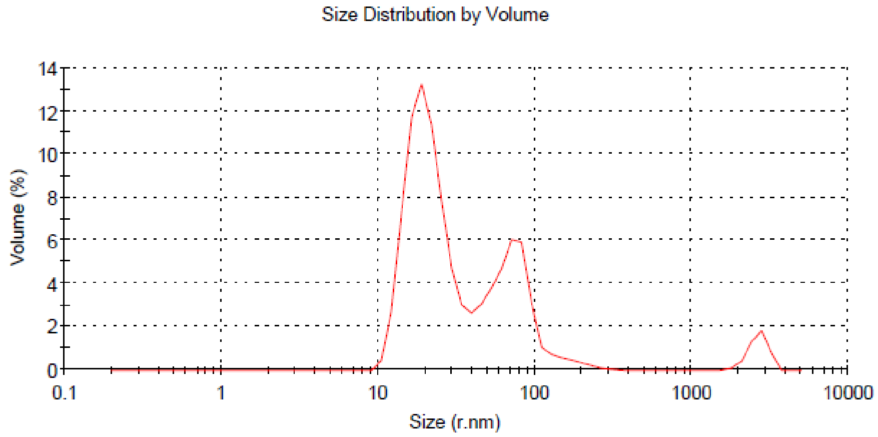

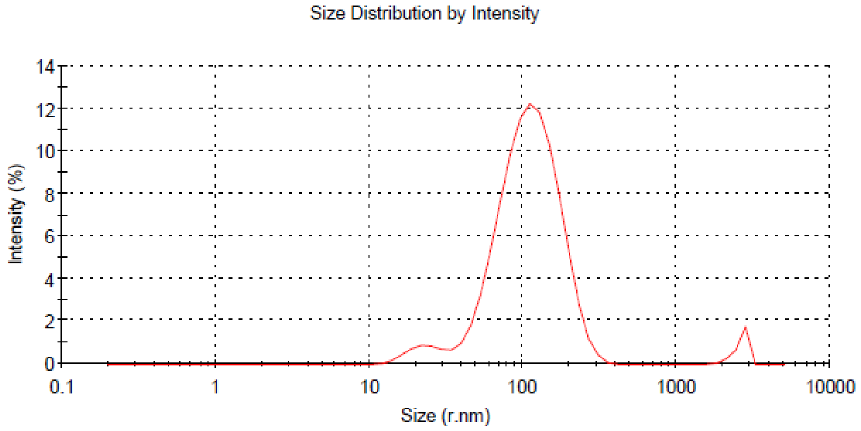

Concentration: 0.4 mgmL^{-1}

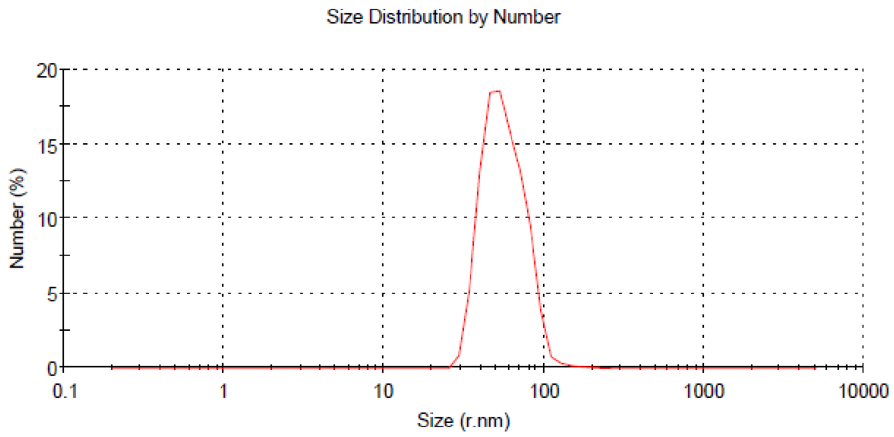

PdI: 0.301

Intercept: 0.942

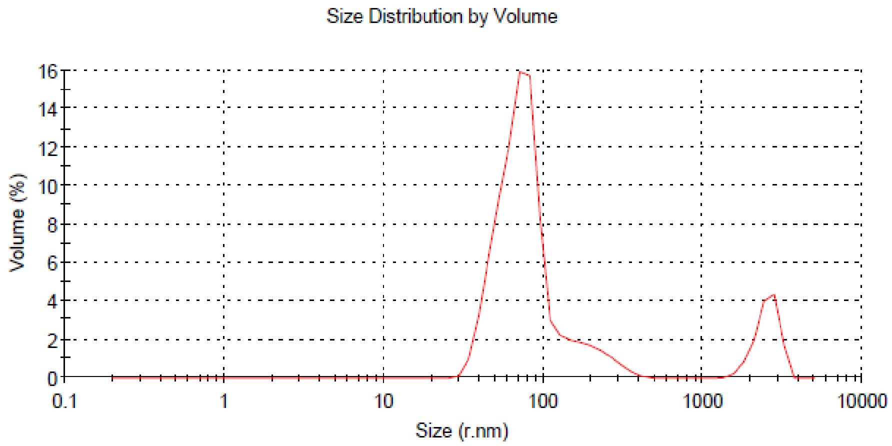

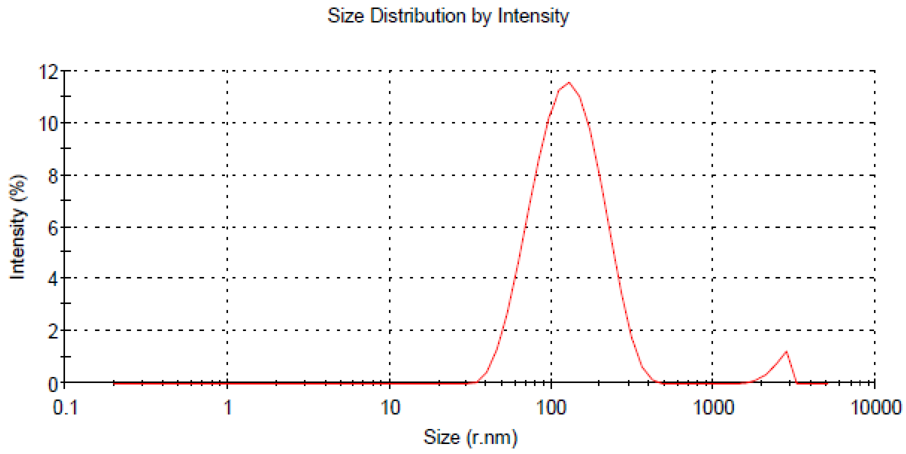

N-Methyl-2(S)-(3′β-acetoxy-17′-chloro-5α-androstan-16-en-2-yl)pyrrolidino[3,4:1,2][60]fullerene (**IIb**)

Concentration: 0.1 mgmL^{-1}

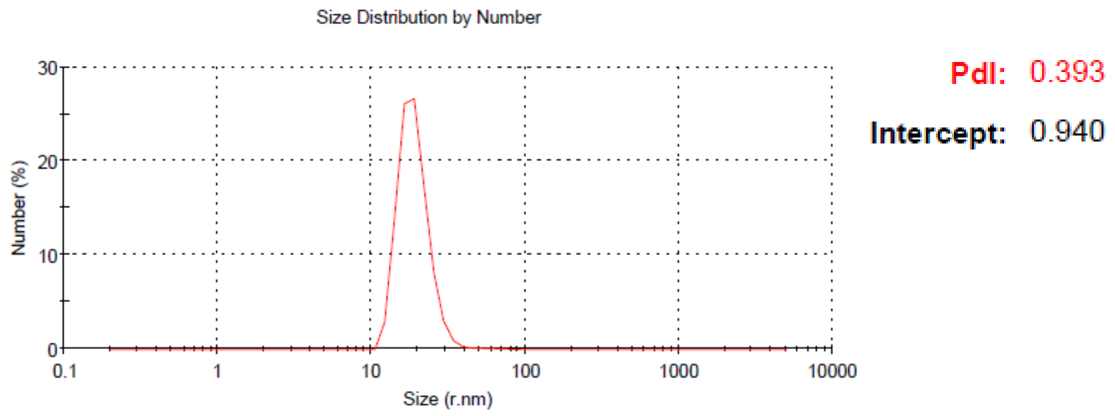

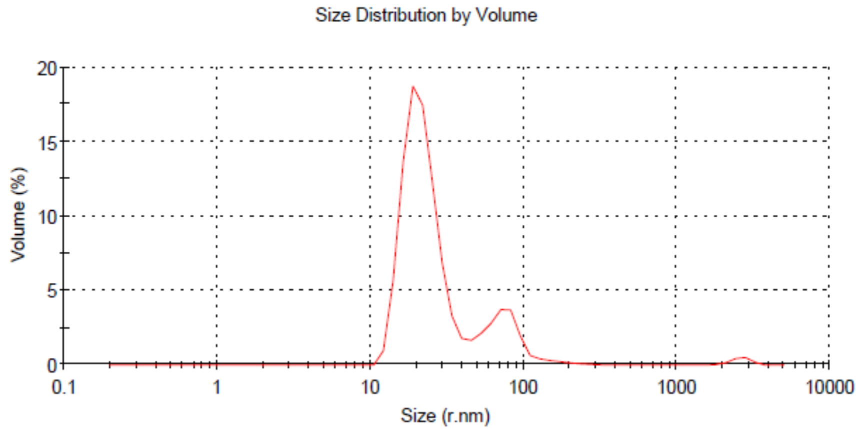

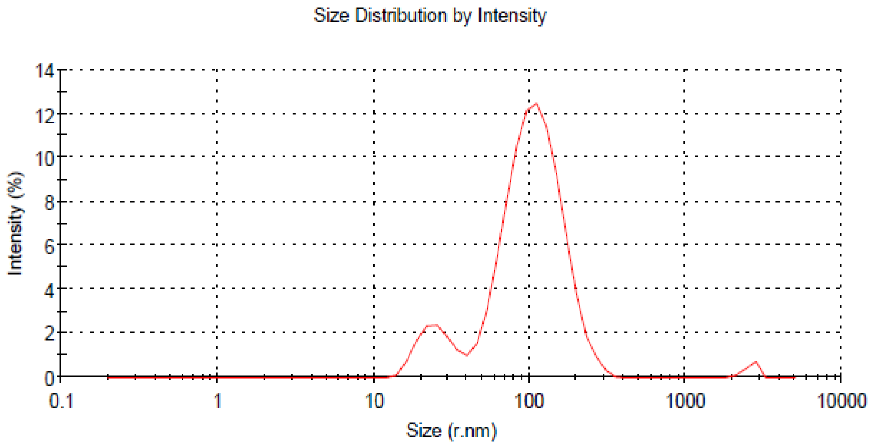

Concentration: 0.4 mgmL^{-1}

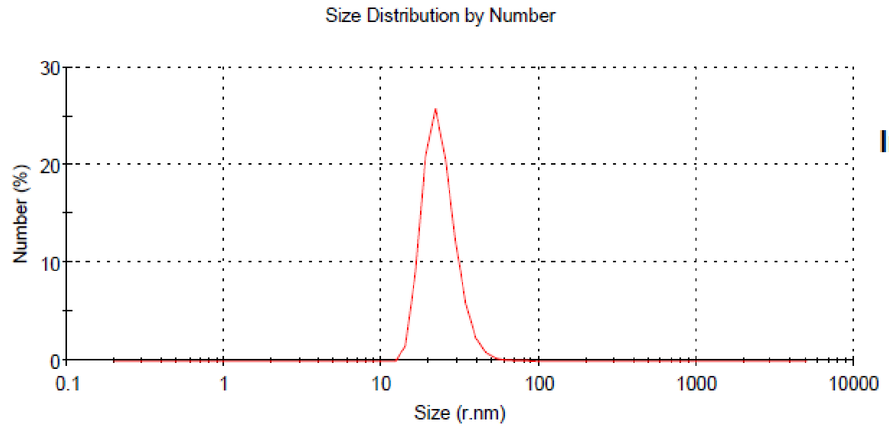

PdI: 0.419

Intercept: 0.925

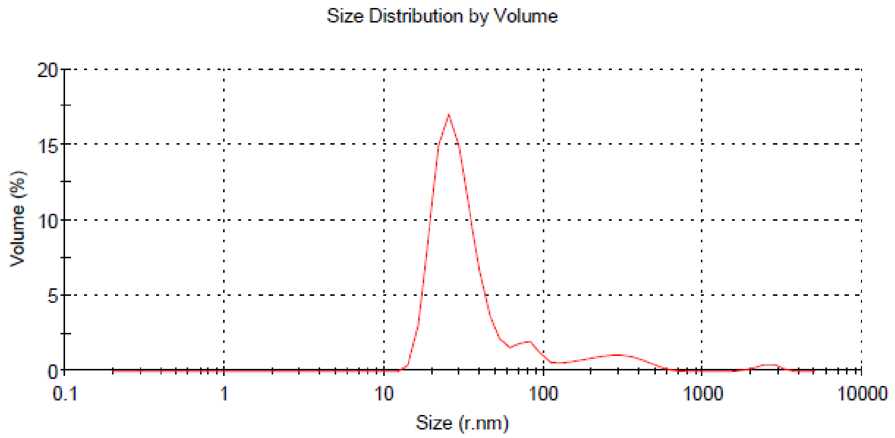

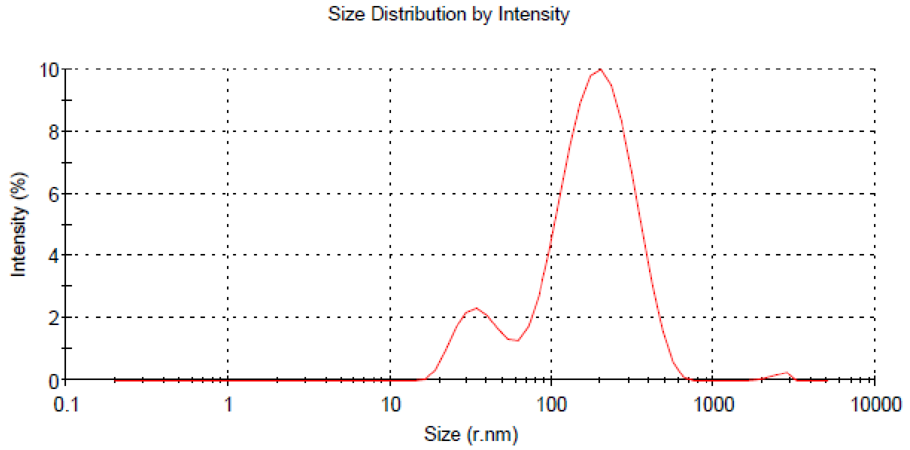

Supplement: File 1 — Additional TEM images and DLS data for the C60–androsterone conjugates Ia,b and IIa,b. [file Beilstein_J_Nanotechnol-05-374-s001.pdf]
